# Application of positron emission tomography imaging to cancer screening

**DOI:** 10.1054/bjoc.2000.1496

**Published:** 2000-12

**Authors:** S Yasuda, M Ide, H Fujii, T Nakahara, Y Mochizuki, W Takahashi, A Shohtsu

**Affiliations:** HIMEDIC Imaging Center at Lake Yamanaka, Hirano, Yamanashi, 401-0502, Japan; Department of Surgery, Tokai University School of Medicine, Isehara, Kanagawa, 259-1193, Japan

**Keywords:** PET, FDG, tumour imaging, glucose metabolism, cancer screening

## Abstract

Whole-body positron emission tomography (PET) with ^18^F-fluorodeoxyglucose (FDG) is a diagnostic modality that can noninvasively survey the entire body and sensitively detect various cancers. In this study, we examined the potential application of whole-body PET for cancer screening in asymptomatic individuals. PET was performed in conjunction with conventional examinations including physical examination, laboratory study, ultrasonography and chest computed tomography. Between September 1994 and March 1999, 3165 asymptomatic individuals participated in 5575 screening sessions (2017 men and 1148 women; mean ± SD age, 52.2 ± 10.4 years). Follow-up periods were no less than 10 months. PET results were compared with the screening outcomes. Within 1 year after screening, malignant tumours were discovered in 67 of the 3165 participants (2.1%). PET findings were true-positive in 36 of the 67 cancers (54%). Most of the 36 patients underwent potentially curative surgery; thus a wide variety of cancers were detected by PET at potentially curable stages. However, PET findings were false-negative in 31 of the 67 patients (46%). 14 of these 31 (45%) were of urological origin. FDG PET imaging has the potential to detect a wide variety of cancers at potentially curable stages. However, PET imaging is not suited to screening test of general population because PET examination involves substantial cost. © 2000 Cancer Research Campaign http://www.bjcancer.com


British Journal of Cancer (2000) 83(12), 1607–1611
© 2000 Cancer Research Campaign
doi: 10.1054/ bjoc.2000.1496, available online at http://www.idealibrary.com on IDEAL®

http://www.bjcancer.com

### Application of positron emission tomography imaging to cancer screening

**S Yasuda[1, 2], M Ide[1], H Fujii[1], T Nakahara[1], Y Mochizuki[1, 2], W Takahashi[1] and A Shohtsu[1]**

[1]HIMEDIC Imaging Center at Lake Yamanaka, Hirano, Yamanashi, 401-0502, Japan; [2]Department of Surgery, Tokai University School of Medicine, Isehara, Kanagawa, 259-1193, Japan

**Summary** Whole-body positron emission tomography (PET) with $^{18}$F-fluorodeoxyglucose (FDG) is a diagnostic modality that can non-invasively survey the entire body and sensitively detect various cancers. In this study, we examined the potential application of whole-body PET for cancer screening in asymptomatic individuals. PET was performed in conjunction with conventional examinations including physical examination, laboratory study, ultrasonography and chest computed tomography. Between September 1994 and March 1999, 3165 asymptomatic individuals participated in 5575 screening sessions (2017 men and 1148 women; mean ± SD age, 52.2 ± 10.4 years). Follow-up periods were no less than 10 months. PET results were compared with the screening outcomes. Within 1 year after screening, malignant tumours were discovered in 67 of the 3165 participants (2.1%). PET findings were true-positive in 36 of the 67 cancers (54%). Most of the 36 patients underwent potentially curative surgery; thus a wide variety of cancers were detected by PET at potentially curable stages. However, PET findings were false-negative in 31 of the 67 patients (46%). 14 of these 31 (45%) were of urological origin. FDG PET imaging has the potential to detect a wide variety of cancers at potentially curable stages. However, PET imaging is not suited to screening test of general population because PET examination involves substantial cost. © 2000 Cancer Research Campaign http://www.bjcancer.com

**Keywords**: PET; FDG; tumour imaging; glucose metabolism; cancer screening

Cancer screening is a major healthcare issue. Screening modalities are constantly changing due to improvements in technology. For example, low-dose computed tomography (CT) has a greater likelihood of detecting lung cancer at an earlier and potentially more curable stage than does conventional chest radiography (Kaneko et al, 1996; Henschke et al, 1999). In breast cancer screening, mammography or ultrasonography is now used instead of just palpation. Studies of several other modalities are underway to elucidate their effectiveness (Kramer et al, 1994).

Positron emission tomography (PET) with $^{18}$F-fluorodeoxyglucose (FDG) has been developed to quantitatively assess local glucose metabolism. Because malignant tumours exhibit increased glucose metabolism, quantification of FDG uptake by PET helps to differentiate between benign and malignant tumours (Rigo et al, 1996), to determine the degree of malignancy (Adler et al, 1991), to evaluate the effectiveness of chemotherapy or radiotherapy (Price and Jones, 1995) and to predict prognosis (Okada et al, 1994; Nakata et al, 1997; Oshida et al, 1998). Since the invention of the whole-body imaging technique (Guerrero et al, 1990), PET has also been used to depict hypermetabolic cancers. PET imaging has been shown to be sensitive enough to detect various cancers including lung, breast, colorectal, pancreatic and head and neck cancer, and malignant lymphoma and melanoma (Conti et al, 1996; Rigo et al, 1996; Delbeke, 1999). It can also be used successfully in patients with unknown primary tumour (Lassen et al, 1999). Thus PET imaging has the potential to detect cancers of many types at early stages with a single study.

*Received 9 February 2000*
*Revised 7 August 2000*
*Accepted 15 August 2000*

*Correspondence to:* S Yasuda

Our facility has 3 whole-body PET scanners, and we have begun to use them in addition to conventional modalities for cancer screening (Yasuda and Shohtsu, 1997). In this study, we analysed whether PET imaging is reliably applicable to cancer screening in asymptomatic individuals.

### SUBJECTS AND METHODS

Our institution is a membership-based medical club offering periodic health checks to members. Subjects of this study were club members. As part of our cancer screening programme, whole-body PET scanning was performed in conjunction with conventional modalities between September 1994 and March 1999: chest radiography and spiral CT scan for lung cancer; physical examination and ultrasonography (beginning January 1996) for breast cancer; immunochemical faecal occult-blood test for colorectal cancer; digital examination and serum prostate-specific antigen (PSA) measurement for prostatic cancer; and ultrasonography for thyroid (beginning January 1996), liver and renal cancers. During this period, 3165 asymptomatic club members (2017 men and 1148 women; ages 52.2 ± 10.4 years, mean ± SD) underwent cancer screening including PET for a total of 5575 examinations. All participants fasted for at least 4 h before PET studies. 45 to 60 min after the administration of 260 to 370 MBq FDG, emission scanning was performed with an ECAT EXACT 47 whole-body PET scanner (Siemens/CTI, Knoxville, TN, USA). Images were obtained from the pelvis to the maxilla for 7 min at each bed position. Transmission scanning for attenuation correction was not carried out. Grey-scale hard copy images of transaxial slices were printed and interpreted visually by one physician (SY). Images of coronal and sagittal slices were also available. The criterion for a positive PET finding was a visible, focally-increased FDG uptake that appeared different from physiologic uptake or uptake of well-recognized benign lesions (Engel et al, 1996; Rigo et al, 1996). Results of simultaneously performed conventional examinations were also available. When abnormal findings were noted by conventional examination, the participant was, as a rule, referred to a local hospital for follow-up or further examination. Follow-up periods were no less than 10 months. Follow-up information was obtained directly from study subjects or their doctors, and cancers discovered within 1 year after screening were documented for analysis. Our screening protocols were approved by an independent ethics committee, and informed consent was obtained from each study participant.

### RESULTS

Among our 3165 asymptomatic participants, malignant tumours were identified in 67 (2.1%) within 1 year after screening. PET findings were true-positive in 36 of these cancers (Table 1). Most of the 36 patients underwent successful surgical treatment. 8 of 10 lung cancers were characterized as stage I tumours ($T_1$, tumour 3 cm or less in the greatest dimension; $N_0$, no regional lymph node metastasis; $M_0$, no distant metastasis) (Sobin and Wittekind, 1997). An example PET scan of one such case is shown in Figure 1. 8 participants were found to have thyroid cancer and underwent surgery. Histopathologically, these tumours were determined to be papillary adenocarcinomas, and microscopic lymph node metastasis was observed in 3 patients. The smallest was a nonpalpable 6-mm tumour in which microscopic nodal metastasis was observed in 5 of 37 dissected lymph nodes. 5 patients with breast cancer including the nonpalpable 6-mm cancer underwent potentially curative surgery. The 6-mm breast cancer was not visible on a mammogram obtained later at the patient's local hospital (Yasuda et al, 1999). 4 participants were found to have colorectal cancer and underwent potentially curative surgery. An ovarian cancer and a parapharyngeal cancer (adenoid cystic carcinoma) were included among the remaining 9 PET-positive cancers. Both patients also underwent potentially curative surgery. 4 participants were found to have colonic adenoma with carcinoma in situ and were not counted among the 36 cases.

31 cancers were PET-negative. 22 of these were detected by the conventional examinations performed during the screening (Table 2), and 9 were discovered within 1 year through examinations obtained mainly because of clinical symptoms (Table 3). 14 of these cancers (45%) were of urologic origin. 6 hepatomas, all smaller than or equal to 2 cm in diameter, in 5 participants were false-negative with PET but positive with ultrasonography. Three small cell lung cancers were false-negative with PET: an 8-mm tubular adenocarcinoma and 1.1- and 1.5-cm bronchioloalveolar

**Table 1** PET-positive cancers

| Patient no. | Age | Sex | Diagnosis | Tumour size (cm) | Lymph node metastasis | Treatment | Conventional imaging with positive findings |
|---|---|---|---|---|---|---|---|
| 1 | 66 | M | Lung cancer | 1.0 | – | Surgery | – |
| 2 | 49 | M | Lung cancer | 1.3 | – | Surgery | CT |
| 3 | 55 | F | Lung cancer | 1.3 | – | Surgery | CT |
| 4 | 66 | M | Lung cancer | 1.5 | – | Surgery | CT |
| 5 | 55 | F | Lung cancer | 1.9 | – | Surgery | CR, CT |
| 6 | 65 | M | Lung cancer | 2.4 | – | Surgery | CR, CT |
| 7 | 77 | F | Lung cancer | 2.7 | – | Surgery | CR, CT |
| 8 | 47 | M | Lung cancer | 2.9 | – | Surgery | CR, CT |
| 9 | 67 | M | Lung cancer | 3.6 | – | Surgery | CT |
| 10 | 70 | M | Lung cancer | (Stage III) | (+) | Radiation | CR, CT |
| 11 | 39 | F | Thyroid cancer | 0.6 | + | Surgery | – |
| 12 | 48 | F | Thyroid cancer | 1.0 | – | Surgery | US |
| 13 | 56 | F | Thyroid cancer | 1.0 | + | Surgery | US |
| 14 | 39 | M | Thyroid cancer | 1.1 | + | Surgery | US |
| 15 | 57 | M | Thyroid cancer | 1.1 | – | Surgery | US |
| 16 | 34 | M | Thyroid cancer | 1.2 | – | Surgery | US |
| 17 | 50 | F | Thyroid cancer | 1.4 | – | Surgery | US |
| 18 | 48 | M | Thyroid cancer | 3.0 | – | Surgery | US |
| 19 | 43 | F | Breast cancer | 0.6 | – | Surgery | – |
| 20 | 48 | F | Breast cancer | 1.3 | – | Surgery | US |
| 21 | 44 | F | Breast cancer | 1.8 | + | Surgery | US |
| 22 | 61 | F | Breast cancer | 2.0 | – | Surgery | US |
| 23 | 51 | F | Breast cancer | 2.4 | + | Surgery | US |
| 24 | 73 | M | Colon cancer | 3.4 | + | Surgery | – |
| 25 | 45 | M | Colon cancer | 3.5 | – | Surgery | – |
| 26 | 53 | M | Rectal cancer | 4.0 | – | Surgery | – |
| 27 | 40 | F | Colon cancer | 6.0 | – | Surgery | – |
| 28 | 60 | M | Lymphoma (thyroid) | 6.0 | – | Chemo. + Rad. | US |
| 29 | 52 | F | Lymphoma | NE | NE | Chemotherapy | – |
| 30 | 67 | F | Hepatoma | NE | + | No treatment | US |
| 31 | 76 | M | Metastatic liver cancer | 1.5 | NE | Ethanol injection | US |
| 32 | 64 | M | Parapharyngeal cancer | 4.4 | – | Surgery | – |
| 33 | 36 | M | Gastric cancer | 3.5 | – | Surgery | US |
| 34 | 65 | M | Renal cancer | 4.0 | – | Surgery | US |
| 35 | 71 | F | Ovarian cancer | NE | NE | Surgery | US |
| 36 | 70 | M | Chronic myelogenous leukaemia | NE | NE | Chemotherapy | – |

CR = chest radiography; CT = computed tomography; US = ultrasonography: NE = not evaluated.

*© 2000 Cancer Research Campaign*

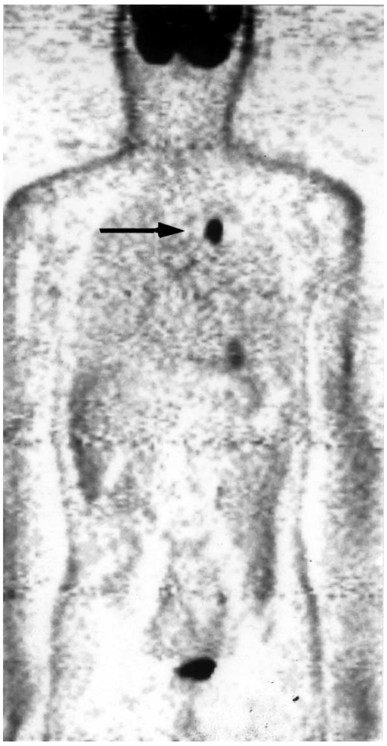

**Figure 1** Lung cancer. On coronal tomographic PET images, high FDG uptake was observed in the left upper lobe of the lung (arrow). Surgery was performed. Histopathologically, the lesion was a 2.4 cm small cell carcinoma. Lymph node metastasis was not observed.

adenocarcinomas. These were also negative with chest radiography but positive with spiral CT. One scirrhous-type breast cancer (stage I, 1.5 cm) was false-negative with PET, though the tumour was palpable and morphologically suspect for malignancy with ultrasonography.

Five participants underwent subsequent surgery due to false-positive interpretation of PET findings. In the early period of this study, we encountered one participant with chronic thyroiditis mimicking a thyroid tumour. Ultrasonographic diagnosis at a local hospital was also of thyroid tumour, and surgery was performed at the hospital. Three participants with benign pulmonary lesions (a 1-cm tuberculoma and two 1-cm organized pneumonias) in whom PET and CT were inconclusive underwent thoracoscopic biopsy (2 cases) or thoracotomy (1 case) for definite diagnoses. One participant was ultimately diagnosed with plasmacyte-rich chronic maxillary sinusitis and underwent surgery at a local hospital (Yasuda et al, 1998).

### DISCUSSION

The ultimate goal of cancer screening is to detect curable cancers that would be fatal if left untreated. However, the biology and natural course of cancers are not fully understood (Hulka, 1998), and the practical goal is reduced mortality. In this respect, the effectiveness of cancer screening has been confirmed with the faecal occult blood test for colorectal cancer (Hardcastle et al, 1996) and mammography for breast cancer in women in their fifth decade (Taubes, 1997). A recent study showed CT to be better than chest radiography for detecting lung cancer (Henschke et al, 1999). Several studies are underway to determine the efficacy of screening for prostate cancer, lung cancer and ovarian cancer (Kramer et al, 1994). These mass screenings target a single organ and do not target low-prevalence cancers. This is due to the lack of a proper modality and cost–benefit considerations.

Whole-body PET imaging can be used to survey the entire body; it is not confined to single organs. In our study, cancers of many organs were positive with PET and were at potentially curable stages. 8 of the 10 PET-positive lung cancers were stage I. The remaining 2 were detected by PET 20 and 23 months prior to final diagnosis at local hospitals. All subjects with PET-positive

**Table 2** Cancers detected by conventional examinations

| Patient no. | Age | Sex | Diagnosis | Clinical stage or tumour size (cm) | Methods of detection |
|---|---|---|---|---|---|
| 1 | 69 | M | Prostatic cancer | Stage A2 | DRE |
| 2 | 52 | M | Prostatic cancer | (Stage B) | PSA |
| 3 | 66 | M | Prostatic cancer | Stage B | PSA |
| 4 | 67 | M | Prostatic cancer | Stage B | PSA |
| 5 | 76 | M | Prostatic cancer | Stage B | PSA |
| 6 | 80 | M | Prostatic cancer | Stage B | PSA |
| 7 | 67 | M | Prostatic cancer | Stage C | PSA |
| 8 | 78 | M | Prostatic cancer | Stage D | PSA |
| 9 | 41 | F | Hepatoma | 1.5 | US |
| 10 | 48 | M | Hepatoma | 1.5 | US |
| 11 | 66 | M | Hepatoma | 1.6 | US |
| 12 | 71 | F | Hepatoma | 1.0, 2.0 | US |
| 13 | 41 | M | Renal cancer | 1.5 | US |
| 14 | 54 | M | Renal cancer | 6.0 | US |
| 15 | 46 | F | Renal cancer | 8.5 | US |
| 16 | 47 | F | Lung cancer | 0.8 | CT |
| 17 | 51 | F | Lung cancer | 1.1 | CT |
| 18 | 60 | M | Lung cancer | 1.5 | CT |
| 19 | 70 | M | Bladder cancer | 3.0 | US |
| 20 | 64 | M | Bladder cancer | 1.5 | US |
| 21 | 53 | F | Breast cancer | 1.5 | PE, US |
| 22 | 46 | F | Gastric cancer | 4.0 | US |

DRE = digital rectal examination; PSA = prostate-specific antigen; US = ultrasonography; CT = computerized tomography; PE = physical examination.

© 2000 Cancer Research Campaign 

**Table 3** Cancers missed by screening

| Patient no. | Age | Sex | Diagnosis | Clinical stage or tumour size (cm) | Period after screening (months) |
|---|---|---|---|---|---|
| 1 | 45 | M | Gastric cancer | Stage I, 7.0 | 2 |
| 2 | 44 | M | Hepatoma | 1.5 | 3 |
| 3 | 77 | F | Gastric cancer | Stage II, 4.0 | 4 |
| 4 | 77 | M | Bladder cancer | 1.0 | 5 |
| 5 | 66 | M | Bile duct cancer | 2.0 | 7 |
| 6 | 68 | M | Gastric cancer | (Limited in the mucosa) | 7 |
| 7 | 53 | F | Cervical cancer | 1.5 | 8 |
| 8 | 56 | M | Oesophageal cancer | (Limited in the mucosa) | 9 |
| 9 | 60 | M | Basal cell cancer (skin) | 0.8 | 10 |

thyroid, breast or colorectal cancer underwent potentially curative surgery. Furthermore, cancers were found that may have been missed by conventional screening methods (such as the 6-mm breast cancer, the 6-mm thyroid cancer, the 1-cm lung cancer and a parapharyngeal cancer).

In our study of asymptomatic individuals, various PET-negative cancers were noted. These can be categorized into 4 groups: urologic cancers, cancers of low cell density (signet ring cell cancer of the stomach and scirrhous-type breast cancer), small cancers and hypometabolic or FDG-negative cancers (lung cancer and hepatoma). Renal excretion of FDG may hamper the detection of urologic cancers (Miraldi et al, 1998). Low tumour cellularity may result in low FDG accumulation (Higashi et al, 1998). The spacial resolution of the PET machine is approximately 6 mm, and in tumours smaller than twice the resolution, partial-volume effects decrease the sensitivity. As for hypometabolic cancers, it is not known if cancers associated with hypometabolism are biologically less aggressive or more indolent than those associated with hypermetabolism. Some of these limitations have been mentioned in previous studies and should be kept in mind when using PET for cancer detection.

In our study, there were a few cases of gastric cancer, one of the most prevalent cancers in Japan. In our country, gastric cancer screening is common, and many of our subjects had undergone stomach examinations at other institutions. In addition, quite a few participants were found to have thyroid cancer, which is not addressed in conventional screening.

One of the major issues of cancer screening is the balance between cost and benefit. PET examination does involve substantial cost compared to other examinations. In terms of cost–benefit, no strong evidence has been obtained favouring the use of PET for oncology patients except with respect to lung cancer (Robert and Milne, 1999). Consequently, there is no convincing rationale for the use of public funds to support PET screening of the general population. In our institution, each member pays the cost. In the future, if PET screening is deemed useful for properly-selected, high-risk groups, debates around cost–benefit issues may arise. Radiation exposure risks heritable effects and carcinogenesis (Hall, 1994). Heritable effects are, however, unlikely to occur when the screening is applied to individuals beyond their reproductive years. Radiation absorbed doses have been measured in PET studies, and the effective dose equivalent was estimated at $2.4 \times 10^{-2}$ mSv/MBq although an overestimation might have occurred in the calculation (Mejia et al, 1991). We used 260 MBq FDG. Therefore, the value is 6.24 mSv, which is lower than the 7.6 mSv value with spiral CT of the chest (Nishizawa et al, 1995). Our actual value was much lower than the calculated value. Previous studies showed that the bladder is the organ receiving the highest doses, and the absorbed dose to the bladder can be reduced if subjects void at the proper time (Jones et al, 1982; Mejia et al, 1991). Our subjects were instructed to void between 45 and 60 min after the administration of FDG. There is the prospect of further reducing the absorbed dose considerably using 3D data aquisition, with which the amount of FDG used is reduced by one-half.

Hypermetabolism is not specific to cancer. Rigo et al (1996) mentioned in their excellent review that FDG PET is not suited to nonselective screening because of the likelihood of false-positives. Our study is the first to deal with a large number of asymptomatic persons. To avoid false-positive interpretations, we took into account the fact that high FDG uptake is observed in some non-malignant lesions. For example, we noticed in the early phase of this study that diffuse thyroidal FDG uptake was a sign of subclinical chronic thyroiditis (Yasuda et al, 1998). False-positive interpretations that lead to unnecessary biopsy or surgery can be largely avoided if the potential sites and characteristics of nonmalignant lesions are recognized in the interpretation of the PET images. Because one of the disadvantages of cancer screening is false-positive results that demand further unnecessary examination, our study results need to be further analysed with regard to false-positive interpretations.

Limitations of this study include the fact that there may be cancers that were missed by the screening and were not discovered within 1 year after screening. In this respect, both the follow-up periods and the examinations conducted are not sufficient to confirm the true incidence of cancers. Another limitation is that our screening was not conducted by PET only. Conventional examinations were performed simultaneously, and the results were available during interpretion of the PET images.

From our data, we believe that PET imaging has the potential to detect a wide variety of cancers at potentially curable stages in asymptomatic individuals, provided the data is analysed by oncologists or radiologists who are experienced in the interpretation of PET images. However, FDG PET imaging has obvious limitations in the detection of urologic cancers, cancers of low cell density, small cancers and hypometabolic or FDG-negative cancers. Because PET examination does involve substantial cost, FDG PET imaging is not suited to screening test of unselected general population. We expect PET screening to be used in properly-selected, high-risk groups in the future.

### ACKNOWLEDGEMENTS

We express special thanks to the staff of our PET centre: Takahito Seio, MRT, Akira Oota, MRT, Hideki Ishihara, MRT, Yuji Yoshimi,

 © 2000 Cancer Research Campaign

MRT, and Mii Ono, for their professional assistance. We also thank Prof Yutaka Suzuki for his helpful advice.

### REFERENCES

Adler LP, Blair HF, Makley JT, Williams RP, Joyce MJ, Leisure G, Al-Kaisi N and Miraldi F (1991) Noninvasive grading of muscloskeletal tumors using PET. *J Nucl Med* **32**: 1508–1512

Conti PS, Lilien DL, Hawley K, Keppler J, Grafton ST and Bading JR (1996) PET and [$^{18}$F]-FDG in oncology: a clinical update. *Nucl Med Biol* **23**: 717–735

Delbeke D (1999) Oncological application of FDG PET imaging: brain tumors, colorectal cancer, lymphoma and melanoma. *J Nucl Med* **40**: 591–603

Engel H, Steinert H, Buck A, Berthold T, Huch Boni RA and von Schulthless GK (1996) Whole-body PET: physiological and artifactual fluorodeoxyglucose accumulations. *J Nucl Med* **37**: 441–446

Guerrero TM, Hoffman EJ, Dahlbom M, Cutler PD, Hawkins RA and Phelps ME (1990) Characterization of a whole body imaging technique for PET. *IEEE Trans Nucl Sci* 676–680

Hall EJ (1994) *Radiobiology for the Radiologist*. Lippincott: Philadelphia

Hardcastle JD, Chamberlain JO, Robinson MHE, Moss SM, Amar SS, Balfour TW, James PD and Mangham CM (1996) Randomised controlled trial of faecal-occult-blood screening for colorectal cancer. *Lancet* **348**: 1472–1477

Henschke CI, McCauley DI, Yankelevits DF, Naidich DP, McGuinness G, Miettinen OS, Libby DM, Pasmantier MW, Koizumi J, Altorki NK and Smith JP (1999) Early lung cancer action project: overall design and findings from baseline screening. *Lancet* **354**: 99–105

Higashi T, Tamaki N, Torizuka T, Nakamoto Y, Sakahara H, Kimura T, Honda T, Inokuma T, Katsushima S, Ohshio G, Imamura M and Konishi J (1998) FDG uptake, glut-1 glucose transporter and cellularity in human pancreatic tumors. *J Nucl Med* **39**: 1727–1735

Hulka BS (1988) Cancer screening: degree of proof and practical application. *Cancer* **62**: 1776–1780

Jones SC, Alavi A, Christman D, Montanez I, Wolf AP and Reivich M (1982) The radiation dosimetry of 2-[F-18] fluoro-2-deoxy-D-glucose in man. *J Nucl Med* **23**: 613–617

Kramer BS, Gohagan J and Prorok PC (1994) NIH consensus 1994: screening. *Gyne Oncol* **55**: S20–21

Kaneko M, Eguchi K, Ohmatsu H, Kakinuma R, Naruke T, Suemasu K and Moriyama N (1996) Peripheral lung cancer: screening and detection with low-dose spiral CT versus radiography. *Radiology* **201**: 798–802

Lassen U, Daugaard G, Eigtved A, Damgaard K and Friberg L (1999) 18F-FDG whole body positron emission tomography (PET) in patients with unknown primary tumours (UPT). *Eur J Cancer* **35**: 1076–1082

Mejia AA, Nakamura T, Itoh M, Hatazawa J, Matsumoto M and Watanuki S (1991) Estimation of absorbed dose in humans due to intravenous administration of fluorine-18-fluorodeoxyglucose in PET studies. *J Nucl Med* **32**: 699–706

Miraldi F, Vesselle H, Faulhaber PF, Adler L and Leisure GP (1998) Elimination of artifactual accumulation of FDG in PET imaging of colorectal cancer. *Clin Nucl Med* **23**: 3–7

Nakata B, Chung YS, Nishimura S, Nishihara T, Sakurai Y, Sawada T, Okamura T, Kawabe J, Ochi H and Sowa M (1997) $^{18}$F-fluorodeoxyglucose positron emission tomography and the prognosis of patients with pancreatic adenocarcinoma. *Cancer* **79**: 695–699

Nishizawa K, Maruyama R, Takayama M, Iwai K and Furuya Y (1995) Estimation of effective dose from CT examination. *Nippon Acta Radiologica* **55**: 763–768

Okada J, Oonishi H, Yoshikawa K, Itami J, Uno K, Imaseki K and Arimizu N (1994) FDG-PET for predicting the prognosis of malignant lymphoma. *Ann Nucl Med* **8**: 187–191

Oshida M, Uno K, Suzuki M, Nagashima T, Hashimoto H, Yagata H, Shishikura T, Imazeki K and Nakajima N (1998) Predicting the prognosis of breast carcinoma patients with positron emission tomography using 2-deoxy-2-fluoro [$^{18}$F]-D-glucose. *Cancer* **82**: 2227–2234

Price P and Jones T (1995) Can positron emission tomography (PET) be used to detect subclinical response to cancer therapy. *Eur J Cancer* **31A**: 1924–1927

Rigo P, Paulus P, Kaschten BJ, Hustinx R, Bury T, Jerusalem G, Benoit T and Foidart-Willems J (1996) Oncological application of positron emission tomography with fluorine-18 fluorodeoxyglucose. *Eur J Nucl Med* **23**: 1641–1674

Robert G and Milne R (1999) A Delphi study to establish national cost-effectiveness research priorities for positron emission tomography. *Eur J Radiol* **30**: 54–60

Sobin LH and Wittekind Ch (eds) (1997) *UICC TNM Classification of Malignant Tumours*. 5th ed. Wiley-Liss: New York

Taubes G (1997) The breast-screening brawl. *Science* **275**: 1056–1059

Yasuda S and Shohtsu A (1997) Cancer screening with whole-body $^{18}$F-fluorodeoxyglucose positron-emission tomography (letter). *Lancet* **350**: 1819

Yasuda S, Shohtsu A, Ide M, Takagi S, Takahashi W, Suzuki Y and Horiuchi M (1998) Chronic thyroiditis: diffuse uptake of FDG at PET. *Radiology* **207**: 775–778

Yasuda S, Shohtsu A, Ide M, Takagi S, Kijima H and Horiuchi M (1998) Elevated F-18 FDG uptake in plasmacyte-rich chronic maxillary sinusitis. *Clin Nucl Med* **23**: 116–118

Yasuda S, Kubota M, Tajima T, Tajima T, Umemura S, Fujii H, Takahashi W, Ide M and Shohtsu A (1999) A small breast cancer detected by PET. *Jpn J Clin Oncol* **29**: 387–389

*© 2000 Cancer Research Campaign*
